# Sulphur Water in Stone in the Bladder

**Published:** 1852-09

**Authors:** 


### SULPHUR WATER IN STONE IN THE BLADDER.

We received a letter about a year since from our friend Dr. Debow, mentioning some remarkable cures of calculous diseases, said to have been effected by the *Red Sulphur Spring*, of Macon county, Tenn. It occurred to us that it would be premature to make public the communication at the time, and accordingly we requested the doctor to continue his observations, and procure additional facts confirmatory of the alleged powers of the water, or the reverse. We had the pleasure of receiving the following letter from him a few days since, and although not written with reference to publication we believe he will excuse us for giving it to our readers. If the accounts given him are exaggerated, the true merits of the water will be the sooner ascertained by thus publicly calling attention to it.

—

HARTSVILLE, August 27, 1852.

*Professor Yandell*:—Some months ago I addressed you a letter, on the subject of the probable efficacy of the Red Sulphur Spring Water, in certain forms of disease, but especially in regard to its almost marvellous effects upon such as are afflicted with stone in the bladder. I gave you, in my letter, the history of some cases that have come within my knowledge, and asked of you, how I could most certainly arrive at the fact, that the water did possess the solvent properties attributed to it. I have not had an opportunity fully to carry out your suggestions, owing to my own business preventing me from attending the spring as I had intended. I have, however, seen and heard enough to confirm me in the opinion that the water does, by some means, cause those afflicted with gravel or stone, to throw off quantities of detritus, after using it, so that though suffering intensely at the time of visiting the spring, they have in a few weeks left it either entirely cured, or very greatly relieved. Nor have I known one thus afflicted, who has not been materially benefitted by a stay of a week or two at the spring. I received a letter from a friend at the spring a few days ago, in answer to one from me, making inquiry in regard to the number of persons attending the spring this season, affected with gravel or stone, and with what benefit, and am gratified to be able to say to you, that each and every one, amounting to some seventeen, has been cured, or much benefitted by a stay of from two to four weeks. In confirmation of the above, I will state that I was at the spring sometime about the first of July last, and that I deposited in a large vial, 6 oz., two small stones—one of the magnesian phosphate, and the other oxalate of lime, or mulberry calculus. I submitted both of them to the same treatment, to see what, if any, effect would in course of time be wrought upon them. Fresh water was supplied every morning, and no friction employed except such as would necessarily arise from pouring out and pouring in the water. In thirty-five days the magnesian phosphate was entirely dissolved; and in sixty-one days, the oxalate was reduced to a fine powder. A similar result was had last year, which I intended to communicate to you if I did not.

With these facts before us, are we not warranted in saying, that the water does possess solvent properties, and that such as are afflicted with the disease alluded to, would do well to avail themselves of so easy and so effective a remedy?

The spring has been greatly crowded this season. Persons from a great distance have visited it, and in every instance, as I am informed, have left well pleased with the water, and its effects upon them as well as with the proprietors who are worthy gentlemen.

I may be deceived in this matter, but I think I am not. I have not spoken of any other than cases of stone, but I am fully persuaded that the water is equally effectual in other forms of disease, particularly in scrofulous affections and diseases of the skin, &c. &c.

Very Respectfully, ARCHIBALD M. DEBOW.

